# Overlapping action of T_3_ and T_4_ during *Xenopus laevis* development

**DOI:** 10.3389/fendo.2024.1360188

**Published:** 2024-03-11

**Authors:** Alicia Tribondeau, David Du Pasquier, Médine Benchouaia, Corinne Blugeon, Nicolas Buisine, Laurent M. Sachs

**Affiliations:** ^1^ Unité Mixte de Recherche 7221, Département Adaptation du Vivant, Centre National de la Recherche Scientifique, Muséum National d’Histoire Naturelle, Alliance Sorbonne Universités, Paris, France; ^2^ Watchfrog Laboratory, Evry-Courcouronnes, France; ^3^ Genomique ENS, Institut de Biologie de l’ENS (IBENS), Département de Biologie, École Normale Supérieure, Centre National de la Recherche Scientifique (CNRS), Institut National de la Santé et de la Recherche Médicale (INSERM), Universités Paris Sciences & Lettres (PSL), Paris, France

**Keywords:** thyroid hormones, *Xenopus laevis*, transcriptomic, *Xenopus* Eleutheroembryonic Thyroid Assay, cell proliferation

## Abstract

Thyroid hormones are involved in many biological processes such as neurogenesis, metabolism, and development. However, compounds called endocrine disruptors can alter thyroid hormone signaling and induce unwanted effects on human and ecosystems health. Regulatory tests have been developed to detect these compounds but need to be significantly improved by proposing novel endpoints and key events. The *Xenopus* Eleutheroembryonic Thyroid Assay (XETA, OECD test guideline no. 248) is one such test. It is based on *Xenopus laevis* tadpoles, a particularly sensitive model system for studying the physiology and disruption of thyroid hormone signaling: amphibian metamorphosis is a spectacular (thus easy to monitor) life cycle transition governed by thyroid hormones. With a long-term objective of providing novel molecular markers under XETA settings, we propose first to describe the differential effects of thyroid hormones on gene expression, which, surprisingly, are not known. After thyroid hormones exposure (T_3_ or T_4_), whole tadpole RNAs were subjected to transcriptomic analysis. By using standard approaches coupled to system biology, we found similar effects of the two thyroid hormones. They impact the cell cycle and promote the expression of genes involves in cell proliferation. At the level of the whole tadpole, the immune system is also a prime target of thyroid hormone action.

## Introduction

Multicellular organisms evolved complex communication systems to coordinate developmental programs. One such neuroendocrine system, the hypothalamic–pituitary–thyroid (HPT) axis, is a central player in orchestrating body morphogenesis and homeostasis. The axis is composed of brain thyrotropin-releasing hormone (TRH) neurons, pituitary thyrotropes producing thyroid-stimulating hormone (TSH), and the thyroid gland (1). TRH acts *via* its receptor expressed by the pituitary thyrotropes to stimulate the synthesis of pituitary TSH. Following its release, TSH binds its receptor expressed on the thyroid gland to induce the production and release of thyroid hormones (THs) in the bloodstream, mainly thyroxine (T_4_) and to a lesser extent triiodothyronine (T_3_). T_4_, often considered to be the precursor for T_3_, is converted to T_3_ by mono-deiodinases (DIO) at target tissues. Both THs act by binding to ligand-activated transcription factors (thyroid hormone receptors; TR) belonging to the nuclear receptor family (2). THs’ main action is then through regulating gene transcription (3). Finally, THs negatively feedback on the hypothalamic TRH neurons and thyrotropes to maintain proper TH levels.

THs play key roles in vertebrate growth, metabolism, and development by controlling cell proliferation, differentiation, migration, and homeostasis. Disruption of THs signaling increase the risk of adverse effects such as cognitive deficits and metabolic diseases/disorders. Thus, perturbations of the TH axis are of “high concern”, especially given the adverse effects caused by anthropogenic and natural chemicals (4). The concern is amplified due to the many gaps in the understanding of the link between potential endocrine disruptor compounds (EDCs), the mode of action of their adverse effects, and the numerous disruption mechanisms (5).

*Xenopus laevis* is an ideal model organism to test thyroid axis disruption *in vivo* as THs orchestrate tadpole metamorphosis (6), but most of all, THs actions and mode of actions are highly conserved across vertebrates. Indeed, the conservation during vertebrate evolution of the mechanisms underlying these major biological processes regulated by THs is striking from fish and amphibian metamorphoses to egg hatching in sauropsids and birth in mammals (1). In particular for *X. laevis*, early larval stage [7 days post-fertilization at 21°C, Nieuwkoop–Faber stage 45 (7)] are suitable for *in vivo* screening, as at this stage, tadpoles possess all the elements of TH signaling (TRs, DIOs, and transporters) and are competent to respond to TH agonist and antagonist treatment. The model also benefits from the prolific reproduction with more than 1,000 embryos per clutch, the small size of tadpoles at this stage (5 mm at NF45) nicely fitting into multiple well plates and allowing the use of small amounts of test chemicals, and finally, a developmental window where action of TH agonist and antagonist translate into clear phenotypes, as exemplified by nervous system defects resulting from alterations in the balance of neuron versus glial cell population (8). Last but not the least, a *Xenopus*-based assay has been validated by OECD, the Organization for Economic Co-operation and Development: The *Xenopus* Eleutheroembryonic Thyroid Assay (XETA) test guideline no. 248 is a mid-throughput and short-term/fast assay to measure the response of tadpoles (stage NF 45) to potential thyroid active chemicals. The test exploits the transgenic line of *X. laevis, Tg(thibz:eGFP)*, which expresses GFP under the control of a 850-bp regulatory region of the TH/bZIP gene, encoding a leucine zipper transcription factor highly sensitive to TH regulation (9). However, in the absence of complete knowledge on the effect of TH disruption at this developmental window, the test is used to identify thyroid active chemicals but not to demonstrate any potential adverse effects.

In order to fill this gap, we subjected tadpoles to the conventional XETA procedure, but instead of measuring changes in GFP fluorescence, we characterized transcriptome changes by high throughput sequencing of RNA (RNA-seq technology). Transcriptome analysis was chosen because it provides a global and unbiased view of the messenger RNA molecules produced from virtually every gene and specific changes according to cell types, the biological and environmental state of the cells at the time of measurement. This strategy is therefore particularly well suited to monitor THs-induced biological responses because they mainly act through nuclear receptors, in which the transcription factor activity can deeply alter the transcriptome of target cells. Another advantage is that XETA treatment duration is 3 days, thereby providing a relatively late readout of the action of THs, well suited to infer novel end points. In this work, we focus on treatments with agonists, T_3_ and T_4_, the two main natural hormones. This choice is justified by 1) the low level of circulating THs at stage NF45, offering a unique *in vivo* situation to measure agonist effects, and 2) the potential difference in the bioavailability of both T_3_ and T_4_ at target cells due to variation in transport, deiodination, and TR binding. Importantly, the transcriptome analysis was realized on RNA extracted from whole tadpole. Despite the difficulties inherent to any measurement on a whole animal, the long-term goal is to identify and provide novel molecular markers of TH signaling alterations readily transferable into novel endpoints for the XETA test guideline. In this context, and given the current need to screen thousands of chemicals, working with whole embryos (i.e., no tissue dissection) is a strategic advantage.

The present work is a proof of principle where we measured transcriptome alterations following treatment with THs. To our knowledge, such broad measure of TH effects has not been described previously. Our results show a strong transcriptional reprogramming for genes involved in all aspects of cell division, with a potential connection to the immune system.

## Materials and methods

### Solutions preparation

T_3_ and T_4_ stock solutions at 6.51 g L^−1^ and 0.8 g L^−1^ were prepared following the protocol of the OECD test guideline no. 248 (*Xenopus* Eleutheroembryo Thyroid Assay) with 3,3′,5-Triiodo-L-thyronine (Sigma: T6397) and L-Thyroxine (Sigma: T2501) powders and ultrapure water. Then, stock solutions were diluted in Evian water to obtain exposure solution at 3.25 µg L^−1^ for T_3_ and 70 µg L^−1^ for T_4_ containing 0.01% of DMSO (Sigma: D8418). The concentration of T_3_ corresponds to the plasma concentration of this hormone during metamorphosis of *X. laevis* and is known to induce morphological changes and modulation of TH target genes in premetamorphic tadpoles (10). The concentration of T_4_ was chosen to give the same fluorescence induction in XETA as that of T_3_ (data not shown). pHs were always between 6.5 and 8.5 as recommended by the XETA protocol. Exposure solutions were stored at 4°C between daily medium renewal and placed at 21°C 3 h before medium renewal.

### Exposure and sample collection

Adult *Xenopus* wild type and transgenic strains were kept in the facilities of Watchfrog (license number: C 91 228 109). The work was conducted in strict accordance with governmental legislations. All procedures involving animal experimentation were approved by the French Ministry of Research under the DAP number APAFIS No. 36464-20220214115229365 v4. Exposure was done following the OECD XETA test guideline. A total of 10 *X. laevis* THb/ZIP-GFP transgenic line tadpoles at stage NF45 (7) were placed in to a six-well plate with 8 mL of exposure solution. Tadpoles were exposed to control (Evian water and 0.01% DMSO), T_3_ or T_4_ treatments in the dark, at 21°C. The media were renewed every day. After 3 days of exposure, the 10 tadpoles were pooled in a single tube (i.e., 10 tadpoles per treatment) and snap frozen (dry ice and ethanol, 99%). Samples were stored at −75°C. Experiments were repeated three times to obtain three biological replicates.

### RNA extraction and purification

RNA extractions were done in two steps. First, a stainless-steel ball (INOX AISI 304 grade 100 AFBMA) and 500 µL RNAble (Eurobio ref: GEXEXT00-0U) were added in each sample and lysed with tissue lyser II apparatus (QIAGEN, Courtaboeuf, France) at 30 Hz for 1 min. Tubes were transferred on ice, and 100 µL of chloroform was added, homogenized vigorously by hand and put on ice for 5 min. Samples were then centrifugated 12,000 *g* at 4°C during 15 min. Next, 175 µL of AquaPhenol (Q-Biogene: AquaPH01) and 100 µL of chloroform were added to 350 µL of supernatant. Following centrifugation, 12,000 *g* at 4°C during 15 min, 250 µL of supernatant was recovered and mixed with 200 µL of ethanol 70%, 10 s on a vortex. The mix was deposited on purification columns (RNeasy Mini Kit, Qiagen: 74104). RNAs were purified according to the manufacturer’s instructions and eluted with 14 µL of RNAse-free water. RNA concentration was measured with nanodrop, and RNA quality was assayed using the Agilent Bioanalyser with standard procedure (Agilent RNA 6000 Nano, Agilent: 5067-1511).

### RNA sequencing and data processing

Library preparation and Illumina sequencing were performed at the Ecole Normale Supérieure Genomique ENS core facility (Paris, France). Messenger (polyA+) RNAs were purified from 1,000 ng of total RNA using oligo(dT). Libraries were performed using the strand-specific RNA-Seq library preparation stranded mRNA Prep, ligation kit (Illumina) and were multiplexed by five on nine flowcells. A 75-bp read sequencing was performed on a NextSeq 500 (Illumina). A mean of 65 ± 23 million passing Illumina quality filter reads was obtained for each of the nine samples (**Supplementary Table S1**). Quality control of sequencing was checked with FASTQC, and all banks were good quality with a PHRED score > 34. The first 13 bp of all reads were clipped to remove sequencing adaptors that remain and mapped on the *X. laevis* genome v9.2 using BOWTIE 0.12.3 (11) with the following parameters: “-5 15 -l 50 -n 1 -m 1”. Data were transformed into BED format with awk script, and redundant reads were removed by using the SORT and UNIQ UNIX commands. Mapping efficiency was higher than 55% and down to approximately 10% after removal of redundancy, resulting in ≥5 million non-redundant reads mapped. Read count table was computed using INSECTBED v2.25 from the BEDTOOLS toolkit. Differential analysis was performed with DESeq v1.10.1 in parametric mode (estimateDispersions parameters: method=pooled, fitType=parametric), and only genes with pval ≤ 0.05 and |log2FC| ≥ 0.95 were considered as differentially expressed.

### Gene Ontology

Gene Ontology analysis was performed with gProfiler (12). METASCAPE software (13) was used to highlight biological processes in a physical protein–protein interaction network context.

### Biological networks

The network construction is based on KEGG pathways database (Kyoto Encyclopedia of Genes and Genomes database) (14) with JEPETTO cytoscape plugin. All pathways that contain at least one DE gene were merged to form a single network. The resulting network is visualized with CYTOSCAPE v3.8.2 software (15) and layout were computed with “edge weighted spring-embedded” algorithm then adjusted by hand to improve the visualization. Functional categories of KEGG pathways are very diverse (signaling pathway, metabolic pathway…), and the reconstructed network should be referenced as a network of biological pathways where nodes represent gene products and edges are functional interactions between them. Hubs are defined as nodes highly connected (degree ≥ 20).

## Results

### T_3_ and T_4_ massively regulate a limited number of biological processes

**Figure 1:**
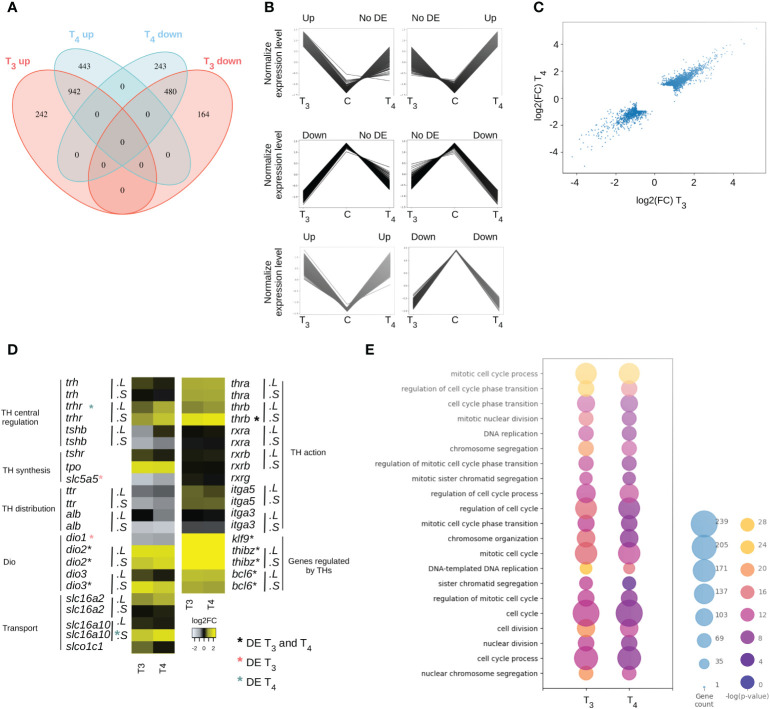
T_3_ and T_4_ treatment induce similar biological responses. **(A)** Differentially expressed (DE) genes up- and downregulated with T_3_ (red) and T_4_ (blue). **(B)** Type of regulation for each DE gene. **(C)** Rate of genes expression level between T_3_ and T_4_ condition. **(D)** Expression level of thyroid signaling related genes. “.L” and “.S”: genes located on long or short chromosome, respectively. *DE genes with both T_3_ and T_4_. *DE genes with T_3_. *DE genes with T_4_. pval ≤ 0.05. **(E)** Biological processes involved in T_3_ and T_4_ responses.

At the end of the differential analysis, 1,828 genes are differentially expressed (DE) following treatment with T_3_ (1,184 upregulated and 644 downregulated) and 2,108 with T_4_ (1,385 upregulated and 723 downregulated) (**Supplementary Tables S2**, **S3**, respectively). The two treatments have 1,422 DE genes in common (**Figure 1A**). The expression of 406 genes is specifically regulated with T_3_ but not T_4_ (242 upregulated and 164 downregulated) and, conversely, 686 genes with T_4_ but not T3 (443 upregulated and 243 downregulated). Overall, two-thirds of the differentially expressed (DE) genes are upregulated following TH treatments. In all cases, the two hormones induce similar responses, either up- or downregulation, as illustrated by the missing synexpression groups displaying opposite regulation following T_3_ and T_4_ treatments (**Figure 1B**). Furthermore, all genes DE for only one of the two hormones still display expression changes albeit not significant (**Figure 1B**), resulting in a good correlation between T_3_ and T_4_ treatment (**Figure 1C**).

The slight differences observed between T_3_ and T_4_ effects can have at least three non-mutually exclusive origins. First, the two hormones have different sets of target genes. Second, they can act on the expression of genes involved in thyroid signaling leading to the modification of TH synthesis, of the biological availability of the hormone in target organs, or of its action. Third, their potency to change gene expression might be different, thus resulting in slightly different regulation kinetics and overlapping but distinct sets of target genes. Overall, all steps of TH signaling are affected by treatment with T_3_ or T_4_ (**Figure 1D**), where gene responses correlate well even though they do not reach statistical significance with both (see above). At the level of the central regulation of TH synthesis, T_3_ and T_4_ not only increase significantly the expression of the thyrotropin receptor (*rtrh*) but also decrease the expression of the β subunit of the thyroid-stimulating hormone (*tshb*), with a stronger effect for T_3_ than for T_4_. At the level of the thyroid gland, both hormones induce an increase in thyroid peroxidase (*tpo*) expression and a significant decrease in the sodium/iodide symporter (*slc5a5*, NIS). For the transport of THs in the blood, a decrease in the expression is observed for transthyretin (*ttr*) and the gene coding for albumin (*alb*). THs also act on the expression of genes involved in the import/export of THs in the cell. The expression of monocarboxylate transporter 8 (*slc16a2*, MCT8) and monocarboxylate transporter 10 (*slc16a10*, MCT10) increases and that of solute carrier organic anion transporter family member 1C1 (*slco1c1*, OATP1c1) increases slightly only under the effect of T_3_. Deiodinases are all significantly regulated by T_3_ or T_4_. The expression of type 1 deiodinase (*dio1*) decreases slightly, that of the two genes encoding type 2 deiodinase (*dio2*) increases and only that of the S form of type 3 deiodinase (*dio3*) increases. Finally, at the level of TH action in target cells, T_3_ and T_4_ increase the expression of the two forms of nuclear receptors (*thra* and *thrb*) and have no effect on their heterodimerization partners (*rxra, rxrb*, and *rxrg*). They also have a weak effect on the expression of the two genes whose products are at the origin of the formation of the membrane receptor (*itga3* and *itga5*). All these observations show that signaling is impacted with both promoting (*trhr*, *tpo*, *alb-like-2*, *dio2*, *thra*, and *thrb*) and limiting effects (*tshb*, *slc5a5*, *ttr*, *alb*, and *dio3*). However, genes known for their TH-modulated expression are well significantly upregulated during T_3_ and T_4_ treatments (*klf9*, *thbzip*, and *bcl6*), with very similar variations for the two hormones (**Figure 1D**).

Next, we carried out gene ontology analysis to highlight the biological functions targeted by T_3_ and T_4_ treatment and their specific action, if any. By taking all the genes expressed (but not necessarily DE) as a reference, the lists of genes DE under the effect of T_3_ or T_4_ are respectively enriched in genes corresponding to 87 and 68 GO terms (**Supplementary Tables S4**, **S5**, respectively). All T_4_ GO terms are found with T_3_, resulting in 19 terms specific to T_3_ (**Supplementary Table S6**). Overall, the GO terms are mainly related to the cell cycle and DNA replication (**Figure 1E**). GO terms only found with T_3_ are related to meiotic processes, G2/M and G1/S phase transition and nuclear division. Then, the same analysis was performed with genes only DE with T_3_ and with T_4_, and no GO terms were significantly enriched.

### Hubs within the biological network are T_3_ and T_4_ target genes

**Figure 2:**
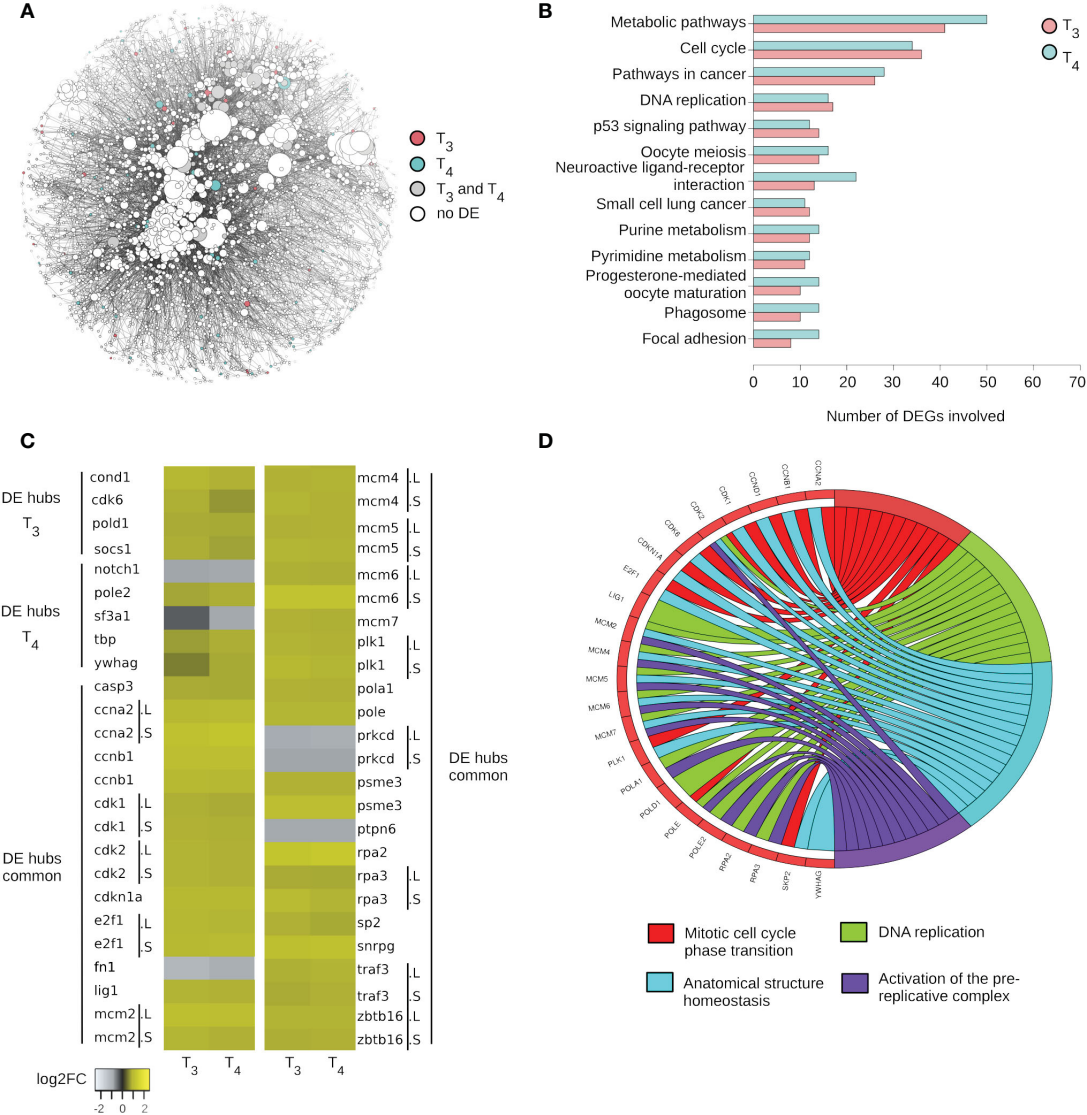
T_3_ and T_4_ impact same pathways and most of DE hubs. **(A)** Network of gene (products) interactions. **(B)** The first 10 KEGG pathways containing most of DE genes in both conditions. **(C)** Expression level of DE hubs. “.L” and “.S”: genes located on long or short chromosome, respectively. **(D)** Biological processes impacted by DE hubs and deducted from a protein–protein interaction analysis.

We next modeled TH response in term of network biology. This integrative approach nicely provides a global and cross-pathway description of their functional impact and help understand the nature of interactions and relations between T_3_ and T_4_ DE genes. Our network coalesced 160 KEGG signaling pathways including at least one DE gene (**Supplementary Table S7**) and is composed of a set of nodes (genes) and a set of undirected connections (edges) representing functional interaction between nodes. The network consists of 16,645 edges and 3,767 nodes (**Figure 2A**). As often the case, only a fraction of DE genes is found in the network: 234 DE genes following treatment with T_3_ and 277 DE genes following treatment with T_4_. This roughly corresponds to approximately a quarter of all DE genes. The KEGG pathways containing the most DE genes are “metabolic pathways” (55), “cell cycle” (39), and “pathways in cancer” (33) (**Figure 2B**, **Supplementary Table S7**).

Structural analysis of networks is commonly used to predict the dynamical properties of biological networks (16). We first focus on one such metric, the degree centrality, corresponding to the number of edges at each node. Most nodes are poorly connected within the network (i.e., low degree). Higher-degree nodes, called hubs, are topologically important and have a strong structural role, which translates into a strong biological importance. This phenomenon, called the centrality–lethality rule, simply refers to the fact that attacking (e.g., knocking down) hubs deeply destabilizes biological networks and results in strong phenotypic alterations. Our network includes 408 hubs (nodes with at least 20 edges), of which 30 are regulated by T_3_ and 31 by T_4_ (**Figure 2C**). The variation in expression for these DE hubs is very similar between the two hormones. There are 26 common hubs between the two treatment conditions (**Figure 2C**): *casp3*, *ccna2*, *ccnb1*, *cdk1*, *cdk2*, *cdkn1a*, *e2f1*, *fn1*, *lig1*, *mcm2*, *mcm4*, *mcm5*, *mcm6*, *mcm7*, *plk1*, *pola1*, *pole*, *prkcd*, *psme3*, *ptpn6*, *rpa2*, *rpa3*, *skp2*, *snrpg*, *traf3*, and *zbtb16*. The majority of them display strong responses and are most often upregulated (log2FC up to 2.23). Many encode for cyclin-dependent kinases (*cdk*), a family of protein kinases with important roles in the control of cell division, while other hubs relate to cell division, such as the components of the minichromosome maintenance protein complex (mcm 2, 4, 5, 6, and 7). This complex forms a DNA helicase essential for genome DNA replication. Several components of the DNA replication machinery, the DNA polymerases subunits *pold1*, *pola1*, and *pole*, are also found in the list of DE hubs. Five hubs are downregulated: *notch1*, *sf3a1*, *fn1*, *prkcd*, and *ptpn6* (**Figure 2C**). *notch1* encodes for a receptor in Notch signaling pathway (NOTCH1, Notch Receptor 1). *sf3a1* encodes for a subunit of the splicing factor complex (SF3A1, splicing factor 3a subunit 1). *prkcd* encodes for a protein kinase C, tumor suppressor, or positive regulator of cell cycle progression. *ptpn6* codes for a protein tyrosine phosphatase. Ontology analysis of DE hubs shows that they are involved not only in mitotic cell cycle phase transition and DNA replication but also in anatomical structure homeostasis and activation of the pre-replicative complex (**Figure 2D**).

### T_3_ and T_4_ differentially expressed genes form specific subnetworks

**Figure 3:**
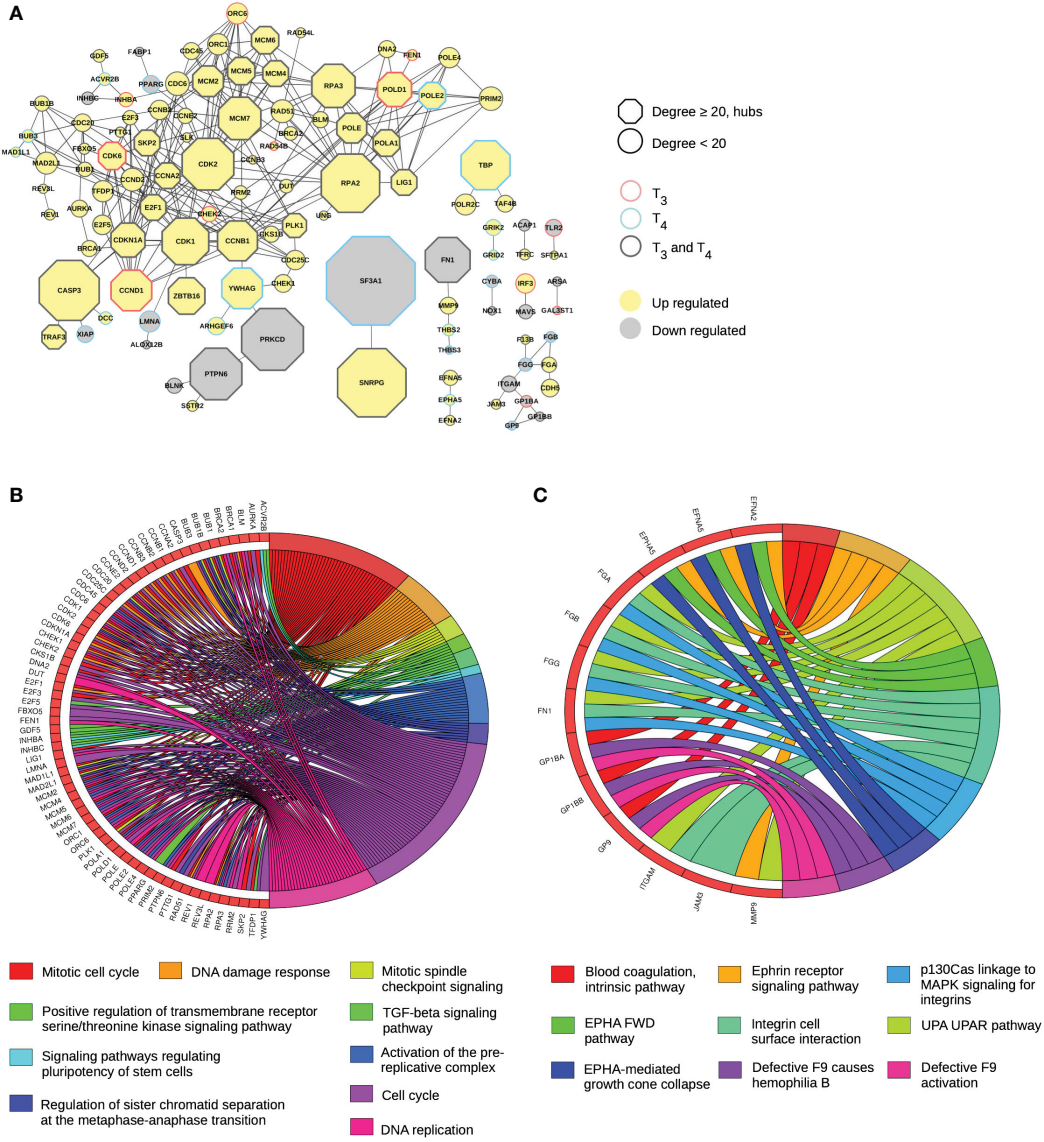
T_3_ and T_4_ responses concentrate in same subnetworks. **(A)** Subnetworks of gene (products) regulated by T_3_ and T_4_. **(B)** Principal biological processes impacted by the huge subnetwork and deducted from a protein–protein interaction analysis. **(C)** Principal biological processes impacted by small subnetworks and deducted from a protein–protein interaction analysis.

Another important feature emerging from of our analysis of molecular networks is a set of “chains” of DE genes, where interacting nodes (as defined in pathways) are collectively DE. As such, chains are *de fact*o hot spots of TH action within the network. We could identify six subnetworks with more than two T_3_ and T_4_ DE genes connected to each other (**Figure 3A**). The largest subnetwork is a giant component of 81 nodes, while the other five are much smaller (size between 2 and 10 nodes). There are also seven pairs of nodes regulated by both T_3_ and T_4_ (**Figure 3A**). Strikingly, of a total of 35 DE hubs, 31 are presently located in the giant component and the remaining four (SF3A1, SNRPG, FN1, and TBP) are located in smaller chains (**Figure 2C**).

Of the 317 T_3_ and T_4_ DE genes found in the entire network, 81 are found in the largest subnetwork, the vast majority of which respond to both T_3_ and T_4_. The transcriptional response of other nodes is relatively similar after treatment with each TH, although it fails to reach statistical significance with one or the other hormone (12 genes for T_3_ and 21 for T_4_). For a large part, genes belonging to this subnetwork relate to cell cycle, DNA replication, and DNA damage response (**Figure 3B**). More than half are involved in mitotic cell cycle (43 out of 81) and cell cycle (58 out of 81). This subnetwork also concentrates 18 upregulated genes coding for protein involved in DNA damage response (BRCA1, CASP3, CCNB1, CCNB2, CCNB3, CCND1, CCND2, CCNE2, CDC25C, CDK1, CDK2, CDK6, CDKN1A, CHEK1, CHEK2, E2F1, RAD51, and RPA2). It is possible to distinguish another subset with four genes (GDF5, ACVR2B, INHBC, and INHBA) involved in TGF-beta signaling pathway. In this subnetwork, the number of downregulated genes is more limited (nine out of 81). They are coding for the proteins BLNK, PTPN6, PRKCD, FABP1, PPARG, INHBC, XIAP, LMNA, and ALOX12B). Two of them have already been sited because they are hubs (PTPN6 and PRKCD). We note that they belong to a chain with BLNK, a protein playing critical role in B-cell development.

Other chains are much smaller. The longest is composed of 10 nodes (F13B, JAM3, FGA, and CDH5 are upregulated, and GP1BB, GP9, FGB, GP1BA, FGG, and ITGAM are downregulated), linked to platelets and blood coagulation (**Figure 3C**). The next chain is very small, only four nodes (FN1, MMP9, THBS2, and THBS3) are involved in cell-to-cell and cell-to-matrix interactions (**Figure 3C**), two of which (the hub FN1 and THBS3) are being downregulated. The fourth subnetwork is composed of the ephrins EFNA5 and EFNA2, and the ephrin receptor EPHA5, all upregulated by THs (**Figure 3C**). The fifth and last chain composed of more than two nodes includes the upregulated genes coding for TBP, POLR2C, and TAF4B involved in transcription.

There are also seven pairs (chains of length 2) of DE genes. Three pairs are involved in immune system functions: the gene coding for the Toll-like Receptor 2 (TLR2), a crucial mediator of innate immunity activation, is linked to SFTPA1, a surfactant protein involved in the defense against pathogen, both upregulated; the downregulated genes CYBA (phagocyte oxidase complex) and the catalytic subunit NOX1; and IRF3 (upregulated) and MAVS (downregulated), important mediators of innate immune response and interferon signaling. Two other pairs are involved in the nervous system: GRIK2 and GRID2 encoding subunits of the glutamate ionotropic receptor, and ARSA and GAL3ST1 involved neuron myelination. The last two pairs are involved in RNA splicing and recycling endosomes, respectively: the hubs SF3A1 (downregulated) and SNRPG (upregulated), followed by ACAP1 (upregulated) and TFRC (downregulated).

## Discussion

Aside from the well-known diversity of TH actions during amphibian metamorphosis (6), early larval development is also a TH-sensitive period commonly used to test thyroid endocrine disruption. Unfortunately, the effects of TH agonists and TH antagonists at this developmental window are only partly described, and available data are very scarce. Our work, based on endocrinology, RNA-seq, and system biology, is a first attempt to elucidate the transcriptional action of both T_3_ and T_4_ in early *X. laevis* larvae. We show that at the level of whole embryos, T_3_ and T_4_ treatments lead to changes in the mRNA levels of components of the TH signaling pathway and a strong transcriptional reprogramming of cell division.

### THs regulate the cell cycle machinery

In *Xenopus*, THs are known to induce cell proliferation during metamorphosis. During intestinal remodeling, adult intestinal stem cells multiply and differentiate into adult epithelial system, while the larval epithelium degenerates (17). Furthermore, transcriptome analysis of intestinal cells from premetamorphic wild-type versus *TRα*-knockout tadpoles treated with or without T_3_ showed strong enrichment with cell-cycle-related genes (18). Similar results were obtained in other tissues such as hindlimbs, which appear during metamorphosis (19), and brain, which is deeply remodeled through neural cell proliferation in ventricular and subventricular zones (20). THs are also important mediators of cell proliferation in mammals (21) for cells as diverse as rat pituitary GH-producing cells, which proliferate in response to T_3_ by shortening G1 phase (22), hepatocytes (23), intestinal epithelial cells (24), cardiomyocytes (25), and skin cells (26).

Here, we show that T_3_ and T_4_ actions induce the expression of many genes involved in cell proliferation, and to this regard, our results are similar to the TH-induced transcriptome reprogramming typically found at metamorphosis in specific tissues (18–20). A significant fraction of them is located in the largest subnetwork, which is involved in most of the biological processes related to cell division: “induction of cell cycle”, “control of cell cycle progression”, “DNA replication”, “activation of the pre-replicative complex”, and “DNA repair and sister chromatin separation”.

A set of DE genes is well known for its major role in cell cycle: transcription factors E2F 1, 3, and 5 (all upregulated) mediate cell proliferation (27), while CDC6, CDC45, CDC25C, and CDC20 are cell division cycle proteins involved in the initiation of DNA replication. The control of cell cycle progression is also ensured by cyclin-dependent kinases (28), such as CDK1, which modulates the centrosome cycle and mitotic onset, promotes G2-M transition, and regulates G1 progress and G1-S transition. The CDK2 kinase triggers the duplication of centrosomes and DNA, promotes the E2F transcriptional program and the initiation of DNA synthesis at the G1-S transition, modulates G2 progression, and controls the timing of entry into mitosis by controlling the subsequent activation of the cyclin B/CDK1 complex. CDKN1A, the cyclin-dependent kinase inhibitor 1A, is involved in TP53-mediated inhibition of cellular proliferation in response to DNA damage. CDK6, the cyclin-dependent kinase 6, is important for cell cycle G1 phase progression and transition to S phase. Cyclin A2 (ccna2) controls the cell cycle at the G1/S (start) and G2/M (mitosis) transitions by acting through the formation of specific serine/threonine protein kinase holoenzyme complexes with the cyclin-dependent protein kinases CDK1 or CDK2 (ccne1). Cyclin B1 (ccnb1) and B2 (ccnb2) are involved in the control of the G2/M (mitosis) transition, and cyclin E2 (ccne2) is a regulator of the late G1 and early S phase. Cyclin D1 (ccnd1, CDK4) is a regulatory subunit of the CDK4–CDK6 complex. Interestingly, cyclin D1 expression is known to increase following T_3_ treatment (22, 23).

Most of the components of the Minichromosome Maintenance Complex (MCM) are also DE (mcm2, mcm4, mcm5, mcm6, and mcm7) (29). Only mcm3 is missing, indicating that the vast majority of them is regulated by THs and further supports their role in promoting cell cycle. This complex forms a replicative helicase essential for DNA replication initiation and elongation. ORC1 and ORC6 are both components of the origin recognition complex (ORC) that binds origins of replication and are required to assemble the pre-replication complex necessary at DNA replication initiation (30). Several DNA polymerase coding genes (pola1, pold1, pole, pole2, and pole4) are DE (31). Actors of DNA repair (32) also increase: RAD51, which plays an important role in homologous strand exchange, a key step in DNA repair through homologous recombination; RAD54B, involved in DNA repair and mitotic recombination; the two BRCA DNA repair associated (BRCA1 and BRCA2) that play a central role in DNA repair by facilitating cellular responses to DNA damage; and CHECK1, a serine/threonine-protein kinase, which is required for checkpoint-mediated cell cycle arrest and activation of DNA repair in response to the presence of DNA damage or un-replicated DNA. CHECK1 is a component of the G2/M checkpoint, phosphorylating and inactivating CDC25C, required for cell cycle arrest in response to DNA damage.

Finally, the expression of genes involved in spindle assembly is also induced: the BUB1 mitotic checkpoint serine/threonine kinase, an essential component of the spindle-assembly checkpoint signaling needed for appropriate chromosome alignment (33), and the Aurora kinase A (AURKA), a mitotic serine/threonine kinase that contributes to the regulation of cell cycle progression *via* its association with the centrosome and the spindle microtubules during mitosis. AURKA has a critical role in various mitotic events including the establishment of mitotic spindle, centrosome duplication, centrosome separation and maturation, chromosomal alignment, spindle assembly checkpoint, and cytokinesis. Overall, TH action during early larval period will target mainly biological processes linked to cell proliferation with its increase.

### TH disruption: potential implications for tadpole development

Our results can help in understanding the potential effects of TH signaling disruption during amphibian larval development. Does the increase in cell proliferation cause an adverse effect? The beginning of the larval period is not well known. Organogenesis is generally complete, and the animal grows slowly. For brain development, it was shown that this period is marked by a low level of neurogenesis due to a dramatic lengthening of the average cell cycle with quiescence progenitor cells poised for reactivation at metamorphosis (34, 35). Obviously, THs (and TR) play a causative role in anuran metamorphosis. However, TR is expressed during the entire larval period, well before metamorphosis, and the rise in THs. Depending upon the presence and absence of TH, TRs can act as both activators and repressors of TH-inducible genes, respectively, TRs have dual functions in frog development (36). As a reminder, TRs function initially as repressors of TH-inducible genes at tadpole stages to prevent premature metamorphosis. Later, TRs act as transcriptional activators of the same genes and activate the metamorphic process. The important point is that the function of unliganded TR before metamorphosis ensures a proper period of tadpole growth. This essential function is also highlighted by TR knockout showing that TRs have a role in the timing of the development with the formation of most adult organs/tissues merely requiring the derepression of TR target genes, while larval tissue degeneration requires liganded TR (37). Even though metamorphosis can be induced precociously by exogenous THs, such treatment does not replicate natural metamorphosis. The potential explanations for this discrepancy are the involvement of other hormones such as glucocorticoids (38) and/or the required signal level for metamorphosis that need to be well controlled over the entire period of the tadpole transformation and need to be adapted to each tissue (39). Thus, inadequate level of THs or agonist will lead to improper TH response gene expression, improper developmental rates, and finally adverse effects. Indeed, THs and EDCs can induce changes in the balance of neuron versus glial cell population by modifying the proportion of cell dividing (8). It is well known that this phenomenon drives to nervous system defect and pathologies (40).

If the effect of THs on cell proliferation is the most obvious, our results provide other avenues for identifying potential adverse effects. First, we found a decrease in mRNA level coding for NOTCH1 (Notch receptor 1), one of the only five hubs in our network downregulated. This receptor in Notch signaling pathway is involved in the development of numerous cell and tissue types across species by regulating interactions between physically adjacent cells (41). Since, NOTCH1 participates in organ formation, tissue function, and repair, disruption of its signaling may cause pathological consequences (42). Two downregulated hubs are present in the large subnetwork including the factors involved in the cell cycle. These two genes code for PRKCD and PTPN6. *prkcd* encodes for protein kinase C delta, a negative regulator of cell cycle progression or positive regulator of cell proliferation. This may look contradictory, but PRKCD in normal condition prevents cell cycle progression (43). The decrease in its expression is therefore consistent with the increase in pro-cell division factors observed in our study. However, in stressful conditions, PRKCD can lose its gatekeeper function at cell cycle checkpoints to stimulate essential cell proliferation. The second downregulated hub, *ptpn6*, codes for a protein tyrosine phosphatase involved in growth, differentiation, the mitotic cycle, and oncogenic transformation (44). The protein is expressed primarily in hematopoietic and epithelial cells, downregulating pathways that promote cell proliferation (45). The downregulation of *ptpn6* in our model agrees with this function. Interestingly, PTPN6 also acts as a negative regulator of receptors involved in immune responses (B-cell antigen receptor, T-cell antigen receptor, and natural killer cell-activating receptor) and cytokine receptors (46). The PTPN6’s role in signaling of the innate and adaptive immune system correlates with its link in our network with another downregulated gene, BLNK and PRKCD, forming a chain of proteins coded by downregulated genes. BLNK, the B-cell linker protein, is involved in B-cell development and its activation (47).

The link of our study with the hematopoietic cells is strengthened by several other observations. First, the subnetwork including F13B, GP1BB, GP9, JAM3, FGB, GP1BA, FGG, FGA, ITGAM, and CDH5 is involved in platelet function and blood clotting (48). Second, in the subnetwork composed of the four nodes (FN1, MMP9, THBS2, and THBS3), *fn1* is one of the downregulated hubs and encodes fibronectin involved in cell adhesion and migration processes like embryogenesis, blood coagulation, host defense, and metastasis (49). There are also THBS2 and THBS3 that belong to the thrombospondin family with anti-angiogenic property (50). Third, the Toll-like receptor 2 (TLR2) is downregulated. TLR2 has a crucial role in pathogen recognition and activation of innate immunity (51). TLR2 is linked to SFTPA1 that is upregulated. SFTPA1 is a surfactant protein important in the defense against pathogen in lungs (52). Finally, the last duo of DE genes includes IRF3 (upregulated) and MAVS (downregulated), where IRF3 (interferon regulatory factor 3) plays an important role in the innate immune response against viruses (53) and MAVS (mitochondrial antiviral signaling protein) is a protein required for activation of transcription factors, which modulate expression of IFN-beta and also contributes to antiviral innate immunity (54). Globally, our results suggest that TH treatment might affect hematopoiesis or hematopoietic function that can lead in part to reduced blood coagulation and reduced immune response.

XETA provides a read out of endocrine response mechanisms, but is not designed to detect adverse effects. Whole transcriptome studies have the potential to fill this gap and provide candidate thyroid-responsive biomarkers and end/points. They are, in turn, the building blocks that link together molecular initiating events to the sequence of key events leading to relevant adverse outcomes, at the organism or population level. In this work, we describe the typical TH response in an experimental setting compliant with XETA. As such, we provide the reference data onto which the impact of EDCs can be fully addressed.

In conclusion, early exposure to THs or EDCs acting as agonist might disrupt proper larval development through increase in cell proliferation and decrease in animal capacity to provide immune response.

## Data availability statement

The data presented in the study are deposited in the Sequence Read Archive repository, accession number PRJNA1044078.

## Ethics statement

The animal study was approved by Comité d’éthique de génopole en expérimentation animale CEEA51. The study was conducted in accordance with the local legislation and institutional requirements.

## Author contributions

AT: Data curation, Investigation, Methodology, Validation, Writing – original draft, Writing – review & editing. DDP: Methodology, Writing – review & editing. MB: Investigation, Writing – review & editing. CB: Investigation, Writing – review & editing. NB: Conceptualization, Investigation, Methodology, Supervision, Writing – review & editing. LS: Conceptualization, Funding acquisition, Project administration, Supervision, Writing – original draft, Writing – review & editing.

## Supplementary material

Supplementary Table 1 Number of sequencing reads and mapping statistics.

Supplementary Table 2 Differential expressed genes with T_3_ treatment (pval ≤ 0.05).

Supplementary Table 3 Differential expressed genes with T_4_ treatment (pval ≤ 0.05).

Supplementary Table 4 Biological processes involved with T_3_.

Supplementary Table 5 Biological processes involved with T_4_.

Supplementary Table 6 Biological processes only involved with T_3_.

Supplementary Table 7 KEGG pathways containing at least one DE gene.
